# Investigating suspected gastrointestinal anthrax: a case-control study in Cayapa village, Abra province, Philippines, March 2017

**DOI:** 10.5365/wpsar.2024.15.1067

**Published:** 2024-10-20

**Authors:** Karen B Lonogan, Alethea De Guzman, Vikki Carr de los Reyes, Ma Nemia Sucaldito, Ferchito Avelino

**Affiliations:** a Field Epidemiology Training Program, Department of Health Center for Health Development – Cordillera Administrative Region, Baguio City, Benguet, Philippines.; b Department of Health, Manila, Philippines.

## Abstract

**Objective:**

Due to rising cases of foodborne illness in Cayapa village, Abra province, Philippines, a team was dispatched on 21 March 2017 to conduct an epidemiological investigation. The objectives were to confirm the diagnosis, determine the existence of an outbreak, identify risk factors and recommend prevention and control measures.

**Methods:**

A 1:2 case-control study was conducted. We defined a suspected case as a previously well village resident who developed abdominal pain or diarrhoea, and one or more symptoms of fever, vomiting, sore throat, difficulty swallowing or lymphadenopathy between 27 February and 14 March 2017. Confirmed cases were suspected cases who tested positive for *Bacillus anthracis* through bacterial culture or rt-PCR. Serum and soil samples were collected for testing, and an environmental survey and key informant interviews were conducted. Stata version 13 was used for data analysis.

**Results:**

The epidemic curve indicated a point source outbreak for the 29 cases identified. Common signs and symptoms were abdominal pain (26, 90%), fever (16, 55%) and diarrhoea (14, 48%). One case presented with lymphadenopathy. Interviews revealed that a dead carabao had been butchered and sold to the villagers. The 11 serum specimens and five soil samples tested were negative for *B. anthracis*. After multivariable analysis, consumption of the uncooked meat of the carabao was significantly associated with being a case (adjusted odds ratio: 6, 95% CI: 1.7–18.4).

**Discussion:**

This outbreak was most likely associated with the consumption of the carcass of a dead carabao. Educating such farming communities on preventive measures for zoonotic diseases is recommended.

Anthrax is an infectious disease caused by *Bacillus anthracis*, a gram-positive, rod-shaped bacterium that can form spores. It can occur in humans in three forms: pulmonary, cutaneous and gastrointestinal. (*1*) The incubation period ranges from 15 hours to 60 days but is usually 1–7 days. Cutaneous anthrax is the most common form. It typically presents as a papular skin lesion, surrounded by a ring of fluid-filled vesicles. The central papule eventually ulcerates and forms a dark, depressed black eschar. (*2*)

Gastrointestinal anthrax includes fever and chills, nausea and vomiting, diarrhoea or bloody diarrhoea, abdominal pain, sore throat, lymphadenopathy and difficulty swallowing. (*3*) This may progress to shock, coma and death. In most of the reviewed case reports and related articles about gastrointestinal anthrax, the disease has a mortality rate of 25–60%. (*4*, *5*) However, the spectrum of disease may range from no symptoms to death and may not always be severe. (*6*) Consumption of raw or undercooked meat from an infected animal is the most common mode of transmission for gastrointestinal anthrax. (*7*)

In the Philippines, 20 health events related to anthrax were reported to the Department of Health Epidemiology Bureau from January 1999 to July 2024, through the Field Epidemiology Training Program (FETP), the Event-based Surveillance and Response system and the Philippine Integrated Disease Surveillance and Response system. Of the reported events, five (25%) were from the Cordillera Administrative Region. The first report was in May 2010 with 39 suspected cases in Abra province, and the most recent was in October 2023 with five cases of cutaneous anthrax in Kalinga province.

Cayapa is one of 17 villages of Lagangilang municipality in Abra province which, in 2017, had a projected population of 954. (*8*) The main source of livelihood is agricultural farming, including the raising of livestock such as swine, goats and carabaos. On 21 March 2017, a team of FETP fellows was sent to Cayapa village to investigate an increase in reports of foodborne illness. The team’s objectives were to verify the diagnosis, establish the existence of an outbreak, identify risk factors and recommend prevention and control measures. This is the first report of suspected gastrointestinal anthrax in the Philippines.

## Methods

### Epidemiological investigation

Cases began to appear after the meat of a dead carabao was sold in the community. A sex-unmatched, 1:2  case-control study was conducted to test the hypothesis that consumption of by-products of the implicated carabao was the mode of transmission. Medical records from the Rural Health Unit and Abra Provincial Hospital were reviewed to identify cases and controls. Active case finding was also conducted using a structured questionnaire to collect demographic, clinical and food exposure data from the study participants. For cases and controls who were minors, parents or guardians completed the questionnaire in their stead.

Cases were defined as follows. A suspected case was a previously well resident of Cayapa village who developed abdominal pain or diarrhoea and any of the following symptoms including vomiting, sore throat, difficulty swallowing, lymphadenopathy or fever from 26 February to 15 March 2017. A confirmed case was a suspected case who tested positive for *B. anthracis* through bacterial culture or reverse transcription-polymerase chain reaction (rt-PCR). A control was a resident of Cayapa village living in or near the house of a case with no clinically compatible symptoms and who tested negative for *B. anthracis* during the study period.

### Statistical analysis

Data analysis was conducted using Stata version 13. Variables with *P* < 0.05 in bivariate analysis were included in a multivariable logistic regression model. Backward elimination was employed to refine the model by systematically removing variables with the highest *P*-values exceeding 0.05 at each elimination step. Statistically significant variables identified during the multivariable analysis were reported.

### Key informant interviews, environmental investigation and laboratory testing

Local officials and health officers were interviewed to substantiate the information gathered. An ocular survey was also conducted at the site where the carabao was found dead and where it had grazed to gain in-depth insight into the health event.

Serum specimens were collected from the cases and soil samples from the site for laboratory confirmation. The serum specimens were sent to the Research Institute for Tropical Medicine, while soil samples were sent to the Reference Laboratory of the Department of Agriculture, Cagayan Valley Region. Both sets of samples were tested for bacteriological isolation.

## Results

### Descriptive analysis

**Fig. 1:**
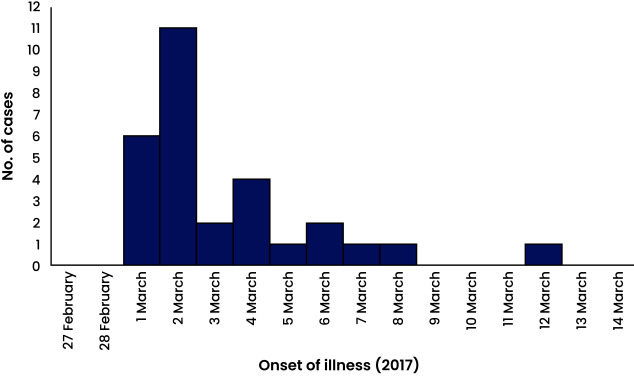
Suspected gastrointestinal anthrax cases by onset of illness (N = 29), Cayapa village, Abra province, Philippines, 27 February–14 March 2017

The first symptomatic cases of suspected gastrointestinal anthrax appeared on the evening of 1 March 2017,  12 hours after the meat of the dead carabao was consumed, and peaked on 2 March (**Fig. 1**). Most of the 29 cases identified were male (15/29, 52%), with ages ranging from 6 to 77 years old (median 17 years). The most affected age group was 5–9-year-olds (6/29, 21%). The most common symptoms were abdominal pain (26/29, 90%), fever (16/29, 55%), diarrhoea (14/29, 48%) and difficulty swallowing (9/29, 31%). One case presented with lymphadenopathy and was further referred to the provincial hospital for management. No deaths were reported.

[**insert** Figure 1]

### Key informant interviews

According to the Municipal Health Officer, the first case from Cayapa village presented on 6 March 2017 with an itchy and painless skin lesion, headache, malaise, difficulty breathing and neck pain that began on 3 March. The case reported that he participated in butchering a dead carabao on 1 March. An interview with the health workers who reported the cases revealed that residents had small blisters that looked like an eschar on their hands. Moreover, since the residents had experienced a similar incident of anthrax in the past, most affected residents were self-medicating with over-the-counter medicines as soon as they felt itchiness on their upper extremities.

The animal owner reported that the carabao looked weak when they purchased it on 28 February 2017 from a nearby municipality. The carabao was taken to a dry, harvested rice paddy to graze. The farm was about 1 km away from the community. On the early morning of 1 March, the animal was found dead, appearing slightly bloated. To recoup the value of the animal, the owner decided to sell the meat. Some parts of the carcass were consumed by those who participated in the butchering of the carabao, while the rest was sold to the community. Some was cooked as a meal on the same day and some was cured or sundried for later cooking. According to the owner, anthrax was never entertained as the cause of death.

The interview with representatives from the local Department of Agriculture revealed that there were three additional animal deaths in the village in the period 8–17 March 2017. All were goats that had died of dehydration and had been grazing in the same area where the dead carabao was found. The goat meat was not consumed as the carcasses were burned and buried. It was also reported that anthrax is endemic in the region.

### Environmental results

During the ocular inspection, it was noted that most of the farm fields were bare and dry. This was consistent with the site where the implicated carabao had grazed and was found dead.

### Laboratory results

The 11 serum specimens collected from cases were negative for any important pathogen, including  *B. anthracis*. The five soil samples collected were positive for *Bacillus cereus* through bacterial culture.

### Analytical study

A total of 58 controls were interviewed. Just over half were males (30/58, 52%), with an age range of 3–80 years old (median 19 years). Univariate analysis revealed that a greater percentage of cases than controls had an occupation associated with handling carabao (48%), ate the uncooked meat of the dead carabao (34%), handled the raw meat (21%), cooked the meat (17%) and helped prepare the meat (3%).

On bivariate analysis, eating the uncooked meat of the dead carabao (odds ratio: 6, 95% CI: 1.5–23.1) was a risk factor for being a case, while multivariable analysis showed that those who consumed the uncooked meat were 6 times more likely to develop signs and symptoms (adjusted odds ratio: 6, 95% CI: 1.7–18.4) than controls (**Table 1**).

**Table 1:** Analysis of factors associated with gastrointestinal anthrax (*n* = 87), Cayapa village, Abra province, Philippines, 27 February–14 March 2017

Factor	Cases (*n* = 29)		Controls (*n* = 58)		Crude odds ratios	Multivariable odds ratios
	No.	%	No.	%	OR (95% CI)	OR (95% CI)
**Male**	**15**	**52**	**30**	**52**	**1 (0.4–2.7)**	**0.8 (0.3–2.2)**
** ≥ 17 years old**	**13**	**45**	**31**	**53**	**0.7 (0.3–1.9)**	**0.3 (0.7–1.0)**
**Handled raw meat of the dead animal**	**6**	**21**	**8**	**14**	**2 (0.4–6.1)**	**3 (0.6–11.0)**
**Cooked meat of the dead animal**	**5**	**17**	**8**	**14**	**1 (0.3–5.1)**	**1 (0.2–3.9)**
**Occupation associated with handling animals**	**14**	**48**	**18**	**31**	**2 (0.7–5.7)**	**3 (0.7–9.4)**
**Ate ≥ 5 tbs of meat of the dead animal**	**18**	**62**	**39**	**67**	**0.8 (0.3–2.3)**	**0.7 (0.2–2.2)**
**Ate uncooked meat of the dead animal**	**10**	**34**	**5**	**9**	**6 (1.5–23.1)**	**6 (1.7–18.4)**

CI: confidence interval; OR: odds ratio; Tbs: tablespoons.

## Discussion

The epidemic curve indicates a point source outbreak of suspected gastrointestinal anthrax in Cayapa village from 1 to 12 March 2017. The clinical manifestations and epidemiological findings suggested that the event could be attributed to the consumption of the by-products from the implicated dead carabao.

One limitation of our study is that *B. anthracis* was not isolated in either the human specimens or soil samples; therefore, the possibility of other foodborne pathogens as the cause of infection cannot be ruled out. The collection of serum specimens from the cases 3 weeks after administration of antibiotics may have contributed to the non-isolation of the bacteria. *B. anthracis* is highly sensitive to antibiotics. Administering antibiotics for more than 24 hours may result in the pathogen not being isolated from cultures taken from any site. (*9*) However, using the Bradford Hill criteria as a framework for epidemiological interpretation of the study, we found temporal, strong statistical and cause-and-effect association between exposure to the dead animal's by-products and the occurrence of disease that is consistent with other published epidemiological studies. (*10*, *11*)

First, the signs and symptoms presented by the cases, including sore throat, neck pain, difficulty swallowing and lymphadenopathy, are distinctive and less commonly associated with typical foodborne illnesses. (*3*) Instead, these are commonly observed among cases of gastrointestinal anthrax. A high level of suspicion of *B. anthracis* as the causative agent cannot be disregarded, especially in regions where anthrax is endemic.

Second, there was a statistical association between eating the uncooked meat of the implicated animal and being a case. This was consistent with other studies on gastrointestinal anthrax. (*12*, *13*)

Third, there was an appropriate time sequence to establish a temporal relationship between the exposure or consumption of the animal’s by-products and the occurrence of disease. All cases had a history of consuming the implicated food before the onset of symptoms. The onset of all cases ranged from 12 hours to 11 days (median 1 day). These fall within the incubation period of gastrointestinal anthrax, which usually ranges between 1 and 7 days but can be as early as < 1 day and extend up to 60 days. (*14*) Also, comparing the incubation period of *B. cereus*, *Staphylococcus aureus* and other common foodborne pathogens with *B. anthracis*, the time sequence is more compatible with the occurrence of gastrointestinal anthrax rather than a typical foodborne illness, which has a very short incubation period. (*13*) Similarly, Maddah, Abdollahi & Katebi (*15*) highlighted the importance of promptly recognizing gastrointestinal anthrax, and that it may be diagnosed based on epidemiological data, such as a history of consuming raw or undercooked livestock products. Bacterial culture or pathologic testing are other ways to diagnose the disease.

The practice of eating and selling the by-products of dead or sick animals has become customary practice in some parts of the Philippines, especially in geographically isolated and disadvantaged areas. This is done to save the value of the dead animal and avoid financial constraints for the owner. However, these practices can lead to a bigger health risk both on the part of the consumer and the producer, as handling and eating sick or dead animals can lead to human infections. Currently, zoonotic diseases are a growing threat to public health and global food security. In March 2014, a highly fatal Henipavirus outbreak was reported in a rural community in the southern province of Sultan Kudarat. Direct exposure, through either contact during slaughtering or eating the meat of the infected animal, was established as the route of infection. (*16*)

Although annual vaccination of livestock against anthrax is highly recommended, particularly in anthrax-endemic areas, it has not been adopted by many countries, especially low- to middle-income countries like the Philippines, due to the cost of vaccination. Hence, educating the public on how zoonotic diseases affect humans and how they are acquired and transmitted can be the most plausible and convenient preventive measure in dealing with similar outbreaks. The use of personal protective equipment when disposing of animal carcasses should be observed, especially if the cause of death is unknown. Also, the prompt notification of sudden animal deaths or the occurrence of similar symptoms to public officials is highly encouraged so that proper investigation and preventive measures can be undertaken by the appropriate authorities. (*6*, *17*, *18*)
